# Knowledge of Oral Cancer among Online Respondent General Dentists: A Cross-sectional Survey

**DOI:** 10.31729/jnma.5651

**Published:** 2021-11-30

**Authors:** Bidhata Ojha, Dipshikha Bajracharya, Radha Baral

**Affiliations:** 1 Department of Oral and Maxillofacial Pathology, Kantipur Dental College and Hospital, Basundhara, Kathmandu, Nepal; 2 Department of Oral and Maxillofacial Pathology, Institute of Medicine, Maharajgunj, Kathmandu, Nepal

**Keywords:** *mortality*, *oral cancer*, *squamous cell carcinoma*

## Abstract

**Introduction:**

Oral cancer is a worldwide medical issue with expanding paces of occurrence and rising mortality rates. Oral cancer is the sixth most common cancer in Nepal with a prevalence of 4.6%. Examination of the oral cavity includes evaluating all teeth, supporting tissue, and surrounding oral tissue and recording the condition of these structures. The present study was conducted to find out the knowledge of oral cancer among general dental practitioners responding to a general survey.

**Methods:**

A cross-sectional survey was conducted among general dental practitioners from March 2020 to July 2020 in 234 sample sizes. Convenience sampling technique was used. Data collection was done after taking ethical approval from the Institutional Review committee (Ref no. 10/020). Data collection was done using a self-administered questionnaire through Google forms. Data analysis was done using Statistical Package of the Social Sciences version 20 software.

**Results:**

Our study showed that most of the participants i.e. 131 (56.2%) have moderately adequate knowledge about oral cancer. One-hundred and eighty four (78.6%) and 178 (76.1%) identified smokeless tobacco and smoking tobacco as high-risk factors for oral cancer. Two-hundred (85.5%) participants recognised buccal/labial mucosa as a common site for oral cancer. And, 138 (59.2%) have knowledge about early detection of oral cancer.

**Conclusions:**

Our study highlights that general dentists have moderate knowledge regarding oral cancer and. Study also states that more practical knowledge and training should be included in undergraduate programs.

## INTRODUCTION

Oral cancer is a worldwide medical issue with expanding paces of occurrence and rising mortality rates.^1^ The incidence of squamous cell carcinoma of the oral cavity varies broadly in different parts of the world.^2^ Oral cancer is the 6^th^ most common cancer in Nepal, with a prevalence of 4.6%.^3^ Despite the oral mucosa being readily accessible for visual inspection, most oral squamous cell carcinomas (OSCCs) are detected during the late stages of the disease, resulting in poor prognosis and patient outcomes.

Examination of the oral cavity includes evaluating all teeth, supporting tissue, and surrounding oral tissue and recording the condition of these structures. A complete oral examination performed by a general practitioner must also include evaluating the oral soft tissues for abnormal color or texture.^4^

This study aims to find out the knowledge among the general dental practitioner about the risk factor of oral cancer and oral potentially malignant disorder (OPMD) and its early detection.

## METHODS

This cross-sectional survey was carried out in Kantipur Dental College, Teaching Hospital. The study was conducted from March 2020 to July 2020. Ethical approval was obtained from the Institutional Review Committee of Kantipur Dental College (reference number: 10/020). Our study population included Nepal Medical Council registered and practicing dental surgeons of Kantipur Dental College and dental surgeons inside Kathmandu Valley. Convenience sampling was done and the sample size was calculated as,

n = Z^2^ × p × q / e^2^

  = 1.96^2^ × 0.5 × 0.5/ (0.07)^2^

  = 196

Where,

n= required sample size,
Z= 1.96 at 95% Confidence Interval (CI),
p= prevalence taken as 50% for maximum sample size,
q= 1-p
e= margin of error, 7%

Taking 10% non-response rate, we arrived at a sample size of 216. But we took 234 participants into the study.

This questionnaire was modified from a study by Rawal et al., and Carter and Ogden et al.^6,7^ Questionnaire was distributed among 250 general dentists for proper response. The questionnaire consisted of four sections and eighteen questions. A self-administered questionnaire was designed, and pilot tested on thirty participants for comprehensibility. Main study did not include results from this test.

Data was entered in Microsoft Excel, and descriptive analysis was done using the Statistical Package for the Social Sciences version 20.

## RESULTS

**Figure 1:**
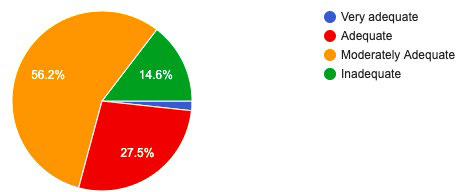
Self-categorization of knowledge by study participants.

A total of 234 general dentists were included in the study. Out of 234 participants 160 (68.5%) were female and 74 (31.5%) were male. The mean age of dental practitioners is 27 years. Among total responses, 131 (56.2%) categorized their knowledge as moderately adequate regarding oral cancer (Figure 1).

**Figure 2:**
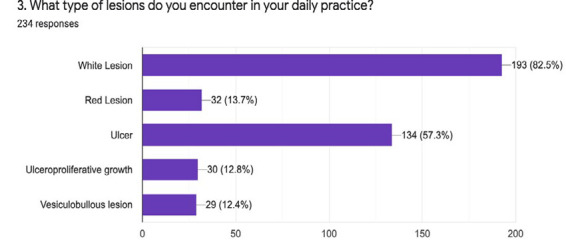
Types of lesions encountered during daily practice.

On asking to have you encountered any potentially malignant lesion, 214 (90.7%) answered yes to the question. Among them, only 59 (27.5%) frequently encountered such lesions, while 155 (72.5%) encountered them rarely. Similarly, among the respondents, most of them 193 (82.5%) encountered white lesions, and 134 (57.3%) encountered ulcers during their daily practice (Figure 2).

**Figure 3:**
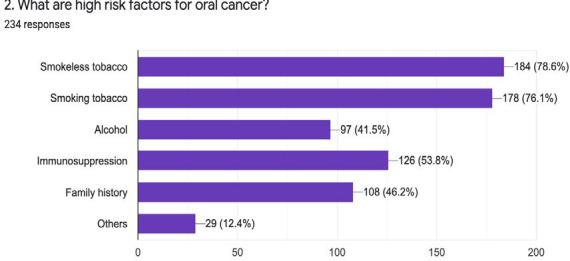
High risk-factors for oral cancer.

**Figure 4:**
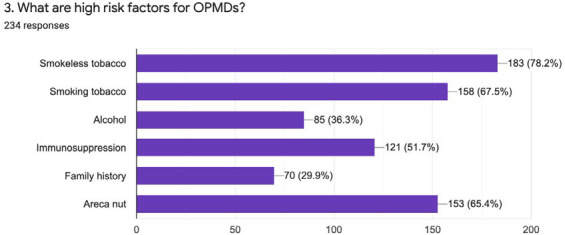
High risk factors for OPMDs.

On asking about OPMDs, most of the respondents, 152 (65%) answered that leukoplakia is OPMDs while 146 (62.4%) answered oral submucous fibrosis. More than 50% of the population answered with oral lichen planus, erythroplakia, and ulcers that haven't healed for more than two weeks. It was evident that 223 (95.2%) general dentists examined the oral mucosa of patients at high risks. Most of the general dentists also mentioned that smoking tobacco, smokeless tobacco, and areca nuts are major high risk agents for an oral lesion (Figures 3, 4).

**Figure 5:**
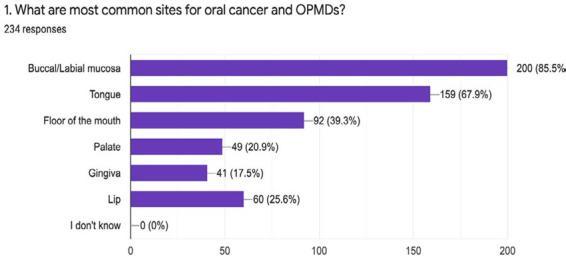
Common sites for oral cancer.

Regarding the sites for oral cancer and OPMDs majority of the respondent 201 (85.8%) answered as buccal/ labial mucosa being the most common site followed by tongue 159 (67.9%) (Figure 5).

One hundred thirty (44.55%) of the total participants preferred to refer the patient to a higher center for those who require biopsy for oral lesions. While very few of them, seven (7.26%) , performed biopsy themselves and 107 (45.72%) called the specialist for the procedure.

Among the total participants, 139 (59.4%) knew the methods for early detection of oral lesions. Among 139 participants who knew about early detection methods, only 64 (38.6%) used specific methods for the early detection of oral lesions. On asking about the different methodologies that they used, only 55 responded to the question. Most of them answered biopsy and oral screening while only seven out of 55 used toluidine blue as an early detective method.

Two hundred and thirty-three (99.5%) participants answered yes to the question regarding counselling on cessation of habits. Similarly, all the participants are interested in getting more knowledge regarding oral cancer and OPMDs. Questions regarding the preference of the method for obtaining more information, multiple choice questions were asked, 141 (60.3%) would like to be involved in related research and would participate in continuing dental education. In contrast, 109 (46.6%) would like to attend the conference to get more information regarding OPMDs and oral cancer.

## DISCUSSION

The present study was carried out to assess the general dentist's knowledge on oral cancer. A thorough oral examination of the oral mucosa leads to early detection and prevention of oral cancer. Oral cancer can be diagnosed at an early stage by visual inspection and some early detecting aid, dentist plays an essential role in doing so.^8^

Our study showed that out of 234 participants 160 (68.5%) were female and 74 (31.5%) were male. Dental education includes theory and practical modality, which is enough for a graduate to examine the oral mucosa of a patient properly. Most of our participants examined oral mucosa whereas eight out of 234 would not examine oral mucosa of all the patients visiting them which states the importance of oral screening on every patient visiting dental hospitals and clinics. A study by Rawal et al. also said that most of their participants examined oral mucosa during their visit to the clinic.^6^

Half of the participants categorize themselves as having moderately adequate knowledge while only five out of 234 participants put themselves in a very adequate category. A study by Kumar et al. also states that the majority of the participants in their study had adequate and up to date knowledge while a similar study by Ahmed et al. and Alaizair et al. stated that most of the participants believe that they don't have enough experience regarding oral lesions which was consistent with our study.^9-11^ This may be due to the varied age groups of the participants and educational modality in various parts of the world.

Knowledge of OPMDs and oral cancer not only includes a screening of oral mucosa, it includes knowledge regarding early detection of the lesions, differentiating such lesions from others as well. Regarding the encounter of OPMDs and oral cancer, 90.7% of the general dentists encountered OPMDs while very few haven't experienced such type of lesions. Among those who encountered OPMDs, maximum number of the participants rarely encountered the lesions and 59 out of 216 participants frequently encountered OPMDs. This experience may be due to the difference in various age groups and the dentist's experience in our study. Different studies have shown that with an increase in experience, dentists develop more clarity about the subject matter.^8,9^ Similarly 82.5% of the participants encountered white lesions during their practice. Among all the 152 participants, leukoplakia was the commonest OPMDs followed by oral submucous fibrosis and oral lichen planus. Data from different studies also state that most general dentists are aware of the common form of OPMDs: leukoplakia and erythroplakia.^8-10^ Regarding the categorization of their knowledge on oral lesions, our study showed diffuse results.

Our study disclosed that 223 out of 234 participants would examine the oral mucosa of the patient at the high-risk group. About the risk factors for oral cancer and OPMDs maximum participants considered smokeless tobacco and smoking tobacco as the major risk factors. Good numbers of the general dentist also considered immunosuppression and areca nut as risk factors for OPMDs and oral cancer. The majority of the participants also warn about the risk factors and their consumptions. Similar results were observed in different studies by Collella et al. and Alaizari et al.^8,10^ Regarding the common sites for OPMDs and oral cancer each of the participants gave their different views. In our study, we found that the most common sites were buccal/labial mucosa but in contrast to this, studies highlight that common sites for OPMDs and oral cancer as the tongue and floor of the mouth.^8,12^

General dentists get training for performing a biopsy and small surgeries during their undergraduate training. On asking them about the practice they perform for the lesions that require a biopsy, only a few of them performed themselves while a high percentage of the general dentist either call a specialist or refer the patients to a higher centre. As in our study, we have discussed that very few participants have categorized themselves as having very few bits of knowledge on oral cancer. This may be the reason that very few dentists perform biopsy themselves. As in other studies, a significant proportion of participating dentists also believed in referring patients to specialists or the higher centers.^10,11^

Having knowledge of diagnosing and treating OPMDs and oral cancer is not enough for a dentist, we can save patients if diagnosed at a very early stage. Different studies showed that the majority of the participating dentists knew about early detection of OPMDs and oral cancer. Most of the dentists perform various methods for early detection of such lesions and early detection also provides a good prognosis. Different tests conducted by a dentist are visual examination, history taking, lymph nodes examination, biopsy, and radiography.^9,10^ Similar views were revealed from our study, along with all these procedures our study also showed that very few participants performed toluidine test for early detection of OPMDs and oral cancer. Toluidine blue test has been established as diagnostic tools in the early detection of OPMDs and oral cancer.^13^

As we have discussed, our study showed smokeless tobacco and smoking tobacco are the major risk factors. Dentists are the individual who can provide counselling on cessation of adverse habits that are responsible for OPMDs and oral cancer. As in our study, 99.5% would like to give counselling on cessation of such habits while authors have reported that participants in their studies also would like to provide tobacco cessation education to their patients. Studies also state that recent graduates are more interested and believe in giving cessation education.^4,10^

It is a well-known fact that continuing dental education optimistically influences dentist's knowledge of OPMDs and oral cancer.^9^ Alaizari, et al. obtained similar results where more than 80% of the participating dentists in their study would like to receive further knowledge on this matter.^10^ Unlike other studies, our study showed slightly different results. All the participants in our study would like to obtain more knowledge in this domain. Among all, most of the participants would like to be involved in related research to upgrade their knowledge, which contrasts with other studies.

As this study was conducted among the general dental practitioners inside Kathmandu valley, it could be conducted with larger sample size outside the valley for a wider range of perception.

## CONCLUSIONS

Similar to other studies, our results also suggest that participating dentists have moderate knowledge regarding early signs and symptoms and major risk factors for oral cancer and OPMDs. Findings also highlight that more practical knowledge must be included in our undergraduate program as well as continuing education programs focusing on oral cancer and its related topic should be given more emphasis.
